# Phenotype-driven protocol switching is associated with improved ART outcomes under constant gonadotropin dosage: a self-controlled analysis of 4,632 cycles

**DOI:** 10.3389/fendo.2026.1816340

**Published:** 2026-05-13

**Authors:** Jie Gao, Yiran Li

**Affiliations:** 1 Shanghai Key Laboratory of Maternal Fetal Medicine, Shanghai Institute of Maternal-Fetal Medicine and Gynecologic Oncology, Shanghai First Maternity and Infant Hospital, School of Medicine, Tongji University, Shanghai, China; 2 Centre for Assisted Reproductive Medicine, Shanghai First Maternal and Infant Hospital, School of Medicine, Tongji University, Shanghai, China

**Keywords:** ART failure, ovarian stimulation, individualized protocol, regression to the mean, cumulative live birth rate

## Abstract

**Background:**

Managing failure after an initial assisted reproductive technology (ART) cycle remains a significant clinical challenge. Clinicians often face a dilemma between repeating the index protocol, escalating gonadotropin (Gn) dosages, or switching to an alternative strategy. We aimed to evaluate whether phenotype-driven protocol switching, specifically addressing issues like over-suppression or poor embryo quality, is more effective than repeating the same protocol.

**Methods:**

This large-scale, retrospective, self-controlled study involved 4,632 patients who underwent two consecutive ART cycles between January 2010 and December 2025. Patients were stratified into four pathways based on their second-cycle strategy: (i) Releasing Suppression (Long-to-Antagonist, n=1,412); (ii) Boosting Quantity (Mild-to-Antagonist, n=220); (iii) Improving Quality (Antagonist-to-Mild, n=829); and (iv) Reference (Antagonist-to-Antagonist, n=2,171). The primary outcome was the cumulative live birth rate (CLBR). Statistical analysis employed the Wilcoxon signed-rank test for paired continuous data and multivariable logistic regression to identify independent predictors of success.

**Results:**

In the Reference group, repeating the same protocol under identical Gn dosages significantly increased the CLBR (9.9% to 51.9%, P < 0.001). Beyond this baseline, individualised switching yielded distinct benefits. The Long-to-Antagonist switch significantly increased the median oocyte retrieval (8.0 [5.0–12.0] vs 9.0 [6.0–13.0], P < 0.001). The Mild-to-Antagonist switch successfully expanded the oocyte cohort in low responders (2.0 [1.0–4.0] vs 3.0 [2.0–5.2], P < 0.001). Conversely, the Antagonist-to-Mild switch did not increase oocyte yield (P = 0.642) but significantly improved the good embryo rate (median 0.0% vs 16.7%, P < 0.001). Multivariable regression confirmed that while age and anti-Müllerian hormone remained dominant prognostic factors, appropriate protocol triage neutralised risks without requiring dose escalation (P > 0.05 for protocol switch strategies).

**Conclusions:**

Phenotype-driven “quantity” or “quality” rescue is associated with significant clinical improvements following ART failure while keeping Gn dosages constant. While tactical switching is effective, a substantial proportion of the success in a second attempt is driven by natural biological variation (regression to the mean).”

## Introduction

Controlled ovarian stimulation (COS) is the fundamental prerequisite for successful Assisted Reproductive Technology (ART), aiming to optimize the recruitment of a follicular cohort to compensate for potential losses during embryogenesis (1). Despite the refinement of individualised stimulation regimens, a significant proportion of patients (up to 40%–50%) encounter failure in their initial cycle due to suboptimal response or compromised embryo quality (2–4). For these patients, the optimal clinical management for a subsequent attempt remains a subject of intense debate: should clinicians persist with the index protocol, escalate the dosage, or transition to an alternative strategy? (5, 6).

In contemporary practice, switching protocols is frequently implemented as an empirical maneuver to overcome prior “resistance” (7). Notably, the GnRH antagonist protocol has emerged as a predominant strategy globally due to its safety profile regarding ovarian hyperstimulation syndrome (OHSS) and patient-friendliness (8). However, when an initial antagonist cycle fails, clinicians often question its intrinsic suitability. While different GnRH analogues offer distinct physiological advantages, current evidence suggests a degree of equivalence in unselected populations, further complicating the rationale for protocol switching (9). A critical limitation of existing literature is the reliance on traditional parallel-group cohort studies. These designs are frequently confounded by substantial inter-individual heterogeneity, masking the specific therapeutic effect of a protocol switch. Crucially, few studies have accounted for regression to the mean (or inter-cycle variability)—the natural tendency for extreme outcomes (such as a failed first cycle) to normalize in a subsequent attempt, independent of any intervention (10). Furthermore, with the maturation of vitrification technology, the CLBR has replaced fresh-cycle outcomes as the most comprehensive indicator of stimulation efficiency (11, 12). Therefore, this retrospective study utilized an extensive dataset from the Reproductive Medicine Centre of Shanghai First Maternity and Infant Hospital. We systematically analyzed a paired cohort of 4,632 patients who underwent two consecutive ART cycles. By stratifying patients into four distinct phenotype-driven transition strategies (Releasing Suppression, Boosting Quantity, Improving Quality, and Reference), we aimed to utilize a self-controlled design to distinguish the true therapeutic benefit of protocol switching from the natural prognosis governed by maternal age and ovarian reserve.

## Materials and methods

### Study design and ethical governance

This large-scale, retrospective, single-centre study employed a self-controlled (paired-cycle) design to facilitate an intra-individual comparison of ovarian stimulation outcomes. The study was conducted at the Reproductive Medicine Centre of Shanghai First Maternity and Infant Hospital, Tongji University School of Medicine. The research protocol was approved by the Institutional Review Board (No: KS25468). Given the retrospective nature of the analysis involving de-identified data, the requirement for informed consent was waived by the IRB.

### Selection criteria and cohort enrollment

**Figure 1:**
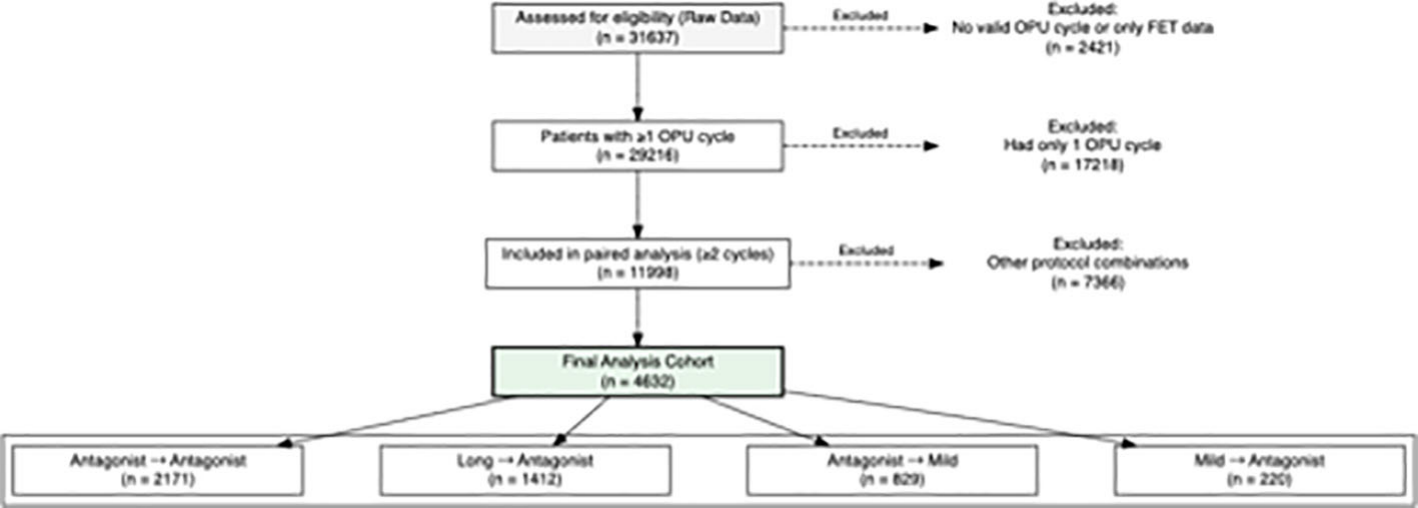
Flowchart of the study population selection. A total of 31,637 fresh cycles were screened, and 4,632 patients who underwent a second cycle after a primary cycle failure were included in the final paired analysis. FOI, failed oocyte identification.

A total of 31,637 ART cycles initiated between January 2010 and December 2025 were screened for eligibility. The inclusion criteria were: (i) women aged 20–45 years; (ii) failure to achieve a live birth in the index cycle (Cycle 1); (iii) undergoing a subsequent stimulation cycle (Cycle 2) at the same institution; and (iv) utilization of standard COS protocols (GnRH antagonist, GnRH agonist, or Mild stimulation). Cycles involving donor gametes, or incomplete medical records were excluded. Following rigorous selection (**Figure 1**), a final cohort of 4,632 patients was enrolled for paired analysis.

### Definitions of failure phenotypes

We standardized the failure phenotypes based on objective clinical thresholds to guide the protocol transition: (1) Recruitment Failure (Quantity Issue): Defined as an oocyte yield of ≤ 3 in the index cycle. (2) Embryo Competence Failure (Quality Issue): Defined as a 0% good-quality embryo rate or abnormal fertilization in the index cycle. These objective thresholds served as the primary clinical triggers for the subsequent ‘triage’ strategy. Consequently, patients exhibiting recruitment failure were predominantly allocated to quantity rescue (e.g., Mild-to-Antagonist), while those with embryo competence failure were directed toward quality rescue (e.g., Antagonist-to-Mild).

### Stratification and transition strategies

To evaluate the efficacy of specific protocol adjustments, patients were stratified into four groups based on the strategy applied in Cycle 2 relative to Cycle 1: 1) Reference Group (Antagonist-to-Antagonist, n=2,171): Patients who repeated the GnRH antagonist protocol, serving as a control to quantify inter-cycle variability. 2) Releasing Suppression (Long-to-Antagonist, n=1,412): Patients switching from a GnRH agonist long protocol to an antagonist protocol. 3) Boosting Quantity (Mild-to-Antagonist, n=220): Patients switching from mild stimulation to an antagonist protocol, which was primarily driven by the need to address recruitment failure. 4) Improving Quality (Antagonist-to-Mild, n=829): Patients switching from an antagonist protocol to mild stimulation, which was primarily driven by the need to address embryo competence failure. Consecutive cycles were defined as two treatment episodes initiated within a 12-month interval. Patients who experienced an interval of more than one year between the two stimulations were excluded to minimize the potential confounding effect of age-related decline in ovarian reserve.

### Stimulation protocols and laboratory procedures

Standardized COS protocols were administered based on maternal age, BMI, and ovarian reserve markers (AMH and AFC). 1) GnRH Antagonist Protocol: Recombinant FSH (rFSH) or human menopausal gonadotropin (hMG) was initiated on Day 2–3 of the cycle. A GnRH antagonist (0.25 mg/day) was introduced when the leading follicle reached 12–14 mm. 2) GnRH Agonist Long Protocol: Pituitary downregulation was achieved using a GnRH agonist starting in the mid-luteal phase of the preceding cycle. 3) Mild Stimulation: Low-dose gonadotrophins (typically ≤150 IU/day) were administered, often in conjunction with oral agents (e.g., clomiphene citrate or letrozole) (13). Oocyte retrieval was performed 34–36 hours after trigger. Fertilization was achieved via conventional IVF or ICSI depending on semen parameters and prior fertilization history. Embryo quality was graded according to the Istanbul Consensus (14, 15). To ensure rigorous self-control, the “constant gonadotropin dosage” in this study was explicitly defined as maintaining an identical starting daily Gn dose between Cycle 1 and Cycle 2, as dictated by our institutional clinical Standard Operating Procedure (SOP). While minor intra-individual variations in total Gn dosage might occur due to slight adjustments in stimulation duration, the consistent starting dose ensures comparable ovarian stimulation intensity across the paired cycles.

### Outcome measures and statistical analysis

The primary endpoint was the CLBR, defined as the delivery of at least one live infant resulting from the fresh or subsequent frozen embryo transfers (FET) derived from the index stimulation cycle. To ensure data completeness and minimize estimation bias, a minimum follow-up period of two years post-oocyte retrieval was applied. Cycles in which patients did not achieve a live birth but still had remaining vitrified embryos at the end of the follow-up period were right-censored and excluded from the CLBR denominator, as their final cumulative outcome remained undetermined. Secondary outcomes included oocyte retrieval number, good embryo rate, and clinical pregnancy rate.

To quantitatively correct and evaluate the effect of natural biological variation (regression to the mean), the absolute increase in CLBR observed in the Reference group (Antagonist-to-Antagonist) was utilized as the baseline proxy for statistical adjustment.

Given the exploratory and retrospective nature of the subgroup analyzes, P-values are reported without formal adjustment for multiple comparisons and should be interpreted as hypothesis-generating. For the multivariable logistic regression, effect sizes are consistently reported as Adjusted Odds Ratios (aOR) with 95% Confidence Intervals (CI). All P-values are two-sided, and P < 0.05 was considered statistically significant.

## Results

### Study population and baseline characteristics

A total of 31,637 ART cycles initiated between January 2010 and December 2025 were screened. Following the selection process, 4,632 patients who failed to achieve a live birth in their index cycle were included for paired-cycle analysis (**Figure 1**).

Baseline characteristics exhibited significant differences across the groups (**Table 1**). The Long-to-Antagonist group was the youngest (mean age 31.8 ± 4.0 years) and had the highest ovarian reserve (AMH 4.1 ± 2.9 ng/mL). In contrast, the Mild-to-Antagonist group represented a poor-prognosis population characterized by advanced age (36.1 ± 5.0 years) and diminished ovarian reserve (AMH 1.2 ± 2.1 ng/mL; Basal FSH 9.8 ± 9.6 IU/L). Analysis of Cycle 1 failure phenotypes revealed that the Mild-to-Antagonist group primarily suffered from recruitment failure (oocyte yield ≤ 3: 70.9%), while the Antagonist-to-Mild group was characterized by compromised embryo quality (zero good embryo rate: 48.4%, **Supplementary Figure 1**).

**Table 1:** Baseline demographic and clinical characteristics of the study population at the first failed cycle.

Characteristic	Long to antagonist N = 1,412	Mild to antagonist N = 220	Antagonist to antagonist N = 2,171	Antagonist to mild N = 829	p-value^1^
Age (years), Mean (SD)	31.8 (4.0)	36.1 (5.0)	34.2 (4.8)	34.8 (5.4)	<0.001
BMI (kg/m²), Mean (SD)	22.2 (3.3)	22.7 (4.3)	22.5 (3.3)	22.5 (3.3)	0.029
Duration of Infertility (years), Mean (SD)	3.2 (2.3)	3.5 (3.4)	2.9 (2.5)	3.3 (2.9)	<0.001
AMH (ng/mL), Mean (SD)	4.1 (2.9)	1.2 (2.1)	3.5 (3.3)	2.7 (3.6)	<0.001
Basal FSH (IU/L), Mean (SD)	6.6 (2.6)	9.8 (9.6)	7.3 (3.4)	8.2 (3.9)	<0.001
Infertility Factor, n (%)					
Tubal factor	588 (42%)	66 (32%)	752 (36%)	300 (38%)	
Male factor	267 (19%)	26 (12%)	313 (15%)	100 (13%)	
Ovulatory disorder	92 (6.6%)	3 (1.4%)	69 (3.3%)	48 (6.0%)	
Endometriosis	50 (3.6%)	5 (2.4%)	30 (1.4%)	16 (2.0%)	
DOR/Advanced Age	7 (0.5%)	42 (20%)	194 (9.4%)	82 (10%)	
Combined factors	74 (5.3%)	20 (9.6%)	283 (14%)	91 (11%)	
Unexplained	91 (6.6%)	5 (2.4%)	128 (6.2%)	32 (4.0%)	
Other	217 (16%)	42 (20%)	304 (15%)	129 (16%)	
Total Gn Dose (Cycle 1) (IU), Mean (SD)	2,396.5 (1,636.0)	1,548.6 (1,196.4)	2,274.2 (1,418.1)	2,348.0 (1,446.4)	<0.001
Duration of Gn (Cycle 1) (days), Mean (SD)	9.9 (4.2)	5.9 (3.8)	7.8 (3.1)	8.1 (3.3)	<0.001

Data are presented as mean (SD) for continuous variables and n (%) for categorical variables. *P*-values were calculated using one-way analysis of variance (ANOVA) for continuous variables and Pearson’s chi-square test for categorical variables to compare differences among the four groups. *P* < 0.05 was considered statistically significant. BMI, body mass index; AMH, anti-Müllerian hormone; FSH, follicle-stimulating hormone; Gn, gonadotropin; SD, standard deviation; DOR, diminished ovarian reserve.

^1^ Kruskal-Wallis rank sum test.

### Ovarian response and laboratory outcomes

Laboratory outcomes in Cycle 2 demonstrated distinct patterns of improvement depending on the transition strategy (**Table 2**).

**Table 2:** Data are presented as median [interquartile range, IQR] for continuous variables and n (%) for categorical variables.

Protocols	Long to antagonist			Mild to antagonist			Antagonist to antagonist			Antagonist to mild		
Outcomes	C1	C2	P	C1	C2	P	C1	C2	P	C1	C2	P
No. of Oocytes Retrieved	8.0 (5.0 - 12.0)	9.0 (6.0 - 13.0)	<0.001	2.0 (1.0 - 4.0)	3.0 (2.0 - 5.2)	<0.001	7.0 (4.0 - 10.0)	7.0 (4.0 - 12.0)	<0.001	4.0 (2.0 - 7.0)	4.0 (2.0 - 7.0)	0.642
No. of 2PN Fertilized	5.0 (3.0 - 8.0)	6.0 (3.0 - 9.0)	<0.001	1.0 (1.0 - 3.0)	2.0 (1.0 - 4.0)	<0.001	4.0 (2.0 - 6.0)	5.0 (3.0 - 8.0)	<0.001	2.0 (1.0 - 4.0)	3.0 (1.0 - 5.0)	<0.001
No. of Good Embryos	1.0 (0.0 - 2.0)	1.0 (0.0 - 3.0)	<0.001	0.0 (0.0 - 1.0)	0.0 (0.0 - 1.0)	<0.001	1.0 (0.0 - 2.0)	1.0 (0.0 - 3.0)	<0.001	0.0 (0.0 - 1.0)	1.0 (0.0 - 2.0)	<0.001
Fertilization Rate (%)	66.7 (42.9 - 85.7)	66.7 (50.0 - 86.7)	<0.001	83.3 (50.0 - 100.0)	80.0 (50.0 - 100.0)	0.210	66.7 (42.9 - 87.5)	71.4 (50.0 - 87.5)	<0.001	66.7 (50.0 - 100.0)	75.0 (53.8 - 100.0)	<0.001
Good Embryo Rate (%)	7.1 (0.0 - 21.4)	16.7 (0.0 - 30.8)	<0.001	0.0 (0.0 - 33.3)	12.5 (0.0 - 33.3)	0.279	12.5 (0.0 - 30.0)	20.0 (0.0 - 37.5)	<0.001	0.0 (0.0 - 25.0)	16.7 (0.0 - 39.6)	<0.001
Blastocyst Formation Rate (%)	33.3 (0.0 - 57.1)	40.0 (17.3 - 60.0)	<0.001	0.0 (0.0 - 61.2)	25.0 (0.0 - 50.0)	0.494	44.4 (0.0 - 73.3)	50.0 (12.5 - 75.0)	<0.001	33.3 (0.0 - 66.7)	41.7 (0.0 - 75.0)	0.038
Clinical Pregnancy Rate	177 (12.5%)	924 (65.4%)	<0.001	11 (5.0%)	92 (41.8%)	<0.001	229 (10.5%)	1145 (52.7%)	<0.001	26 (3.1%)	392 (47.3%)	<0.001
Miscarriage Rate	13 (0.9%)	17 (1.2%)	0.571	1 (0.5%)	1 (0.5%)	1.000	14 (0.6%)	18 (0.8%)	0.556	0 (0.0%)	7 (0.8%)	0.023
Live Birth Rate	164 (11.6%)	907 (64.2%)	<0.001	10 (4.5%)	91 (41.4%)	<0.001	215 (9.9%)	1127 (51.9%)	<0.001	26 (3.1%)	385 (46.4%)	<0.001

P-values represent the intra-individual comparison between Cycle 1 and Cycle 2 within each group. Statistical significance was determined using the Wilcoxon signed-rank test for continuous variables (due to non-normal distribution) and McNemar’s test for binary categorical outcomes (e.g., pregnancy rates). P < 0.05 was considered statistically significant. Note that P-values were not calculated for the distribution of fertilization methods as these represented clinical decisions rather than randomised outcomes. Gn, gonadotrophin; IVF, *in vitro* fertilization; ICSI, intracytoplasmic sperm injection; PGT, preimplantation genetic testing; 2PN, two pronuclei; OHSS, ovarian hyperstimulation syndrome.

Continuous variables are presented as Median (IQR); Categorical variables as n (%).

P-values: Wilcoxon signed-rank test for continuous variables; McNemar’s test for categorical variables.

**Figure 2:**
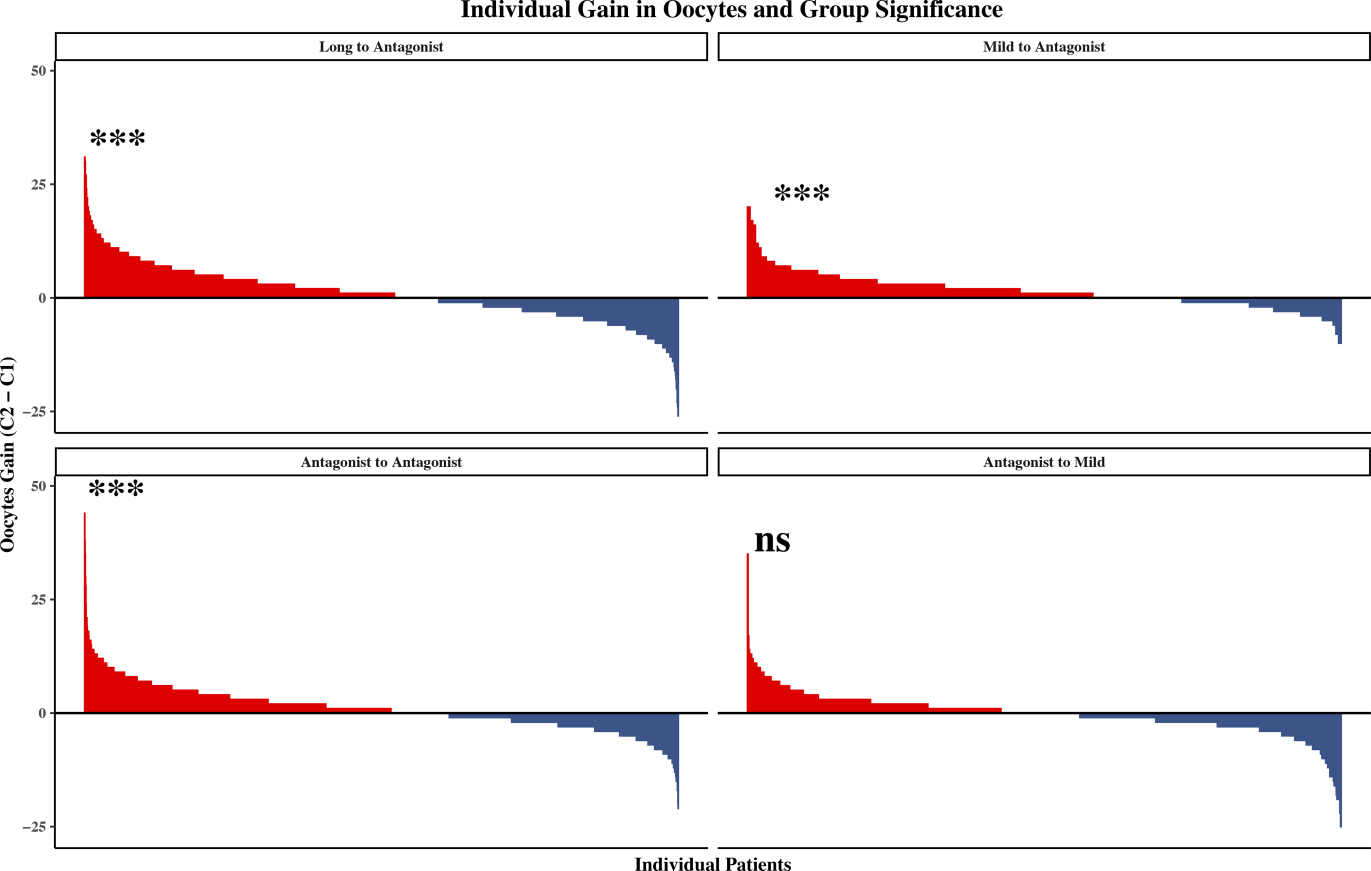
Waterfall plot of individual gain in oocytes retrieved. Each bar represents a single patient. Bars above the zero line indicate an increase in oocyte yield in the second cycle compared to the first. Significant improvements were observed in the Long to Antagonist, Mild to Antagonist, and Antagonist to Antagonist groups. ***P < 0.001; ns, non-significant (Wilcoxon signed-rank test).

**Figure 3:**
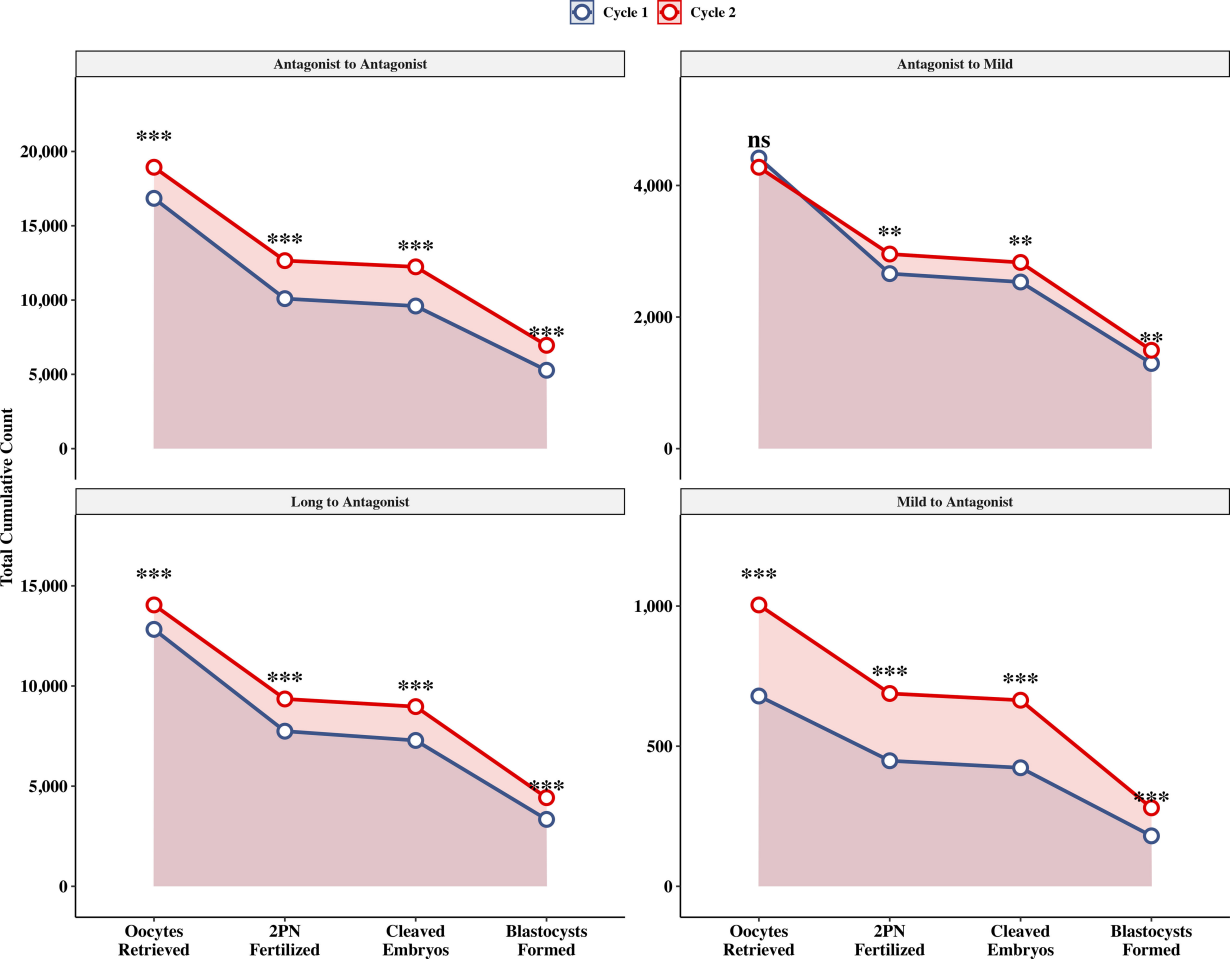
Cumulative attrition funnel of embryo development. The plot illustrates the reduction in embryo loss from oocyte retrieval to blastocyst formation in Cycle 2 (red line) compared to Cycle 1 (blue line). Asterisks indicate significant differences in counts at each developmental stage. ***P < 0.001; **P < 0.01 (Wilcoxon signed-rank test).

The Long-to-Antagonist strategy significantly increased the median number of oocytes retrieved [8.0 (IQR: 5.0–12.0) vs 9.0 (IQR: 6.0–13.0), P < 0.001] and 2PN fertilization [5.0 (3.0–8.0) vs 6.0 (3.0–9.0), P < 0.001]. The most substantial relative gain in recruitment was observed in the Mild-to-Antagonist group, where the median oocyte yield expanded from 2.0 (1.0–4.0) to 3.0 (2.0–5.2) (P < 0.001). Waterfall plots further confirmed a positive net gain in oocytes for the majority of patients in these groups (**Figure 2**). Significant improvements were also observed across all developmental stages (**Figure 3**).

**Figure 4:**
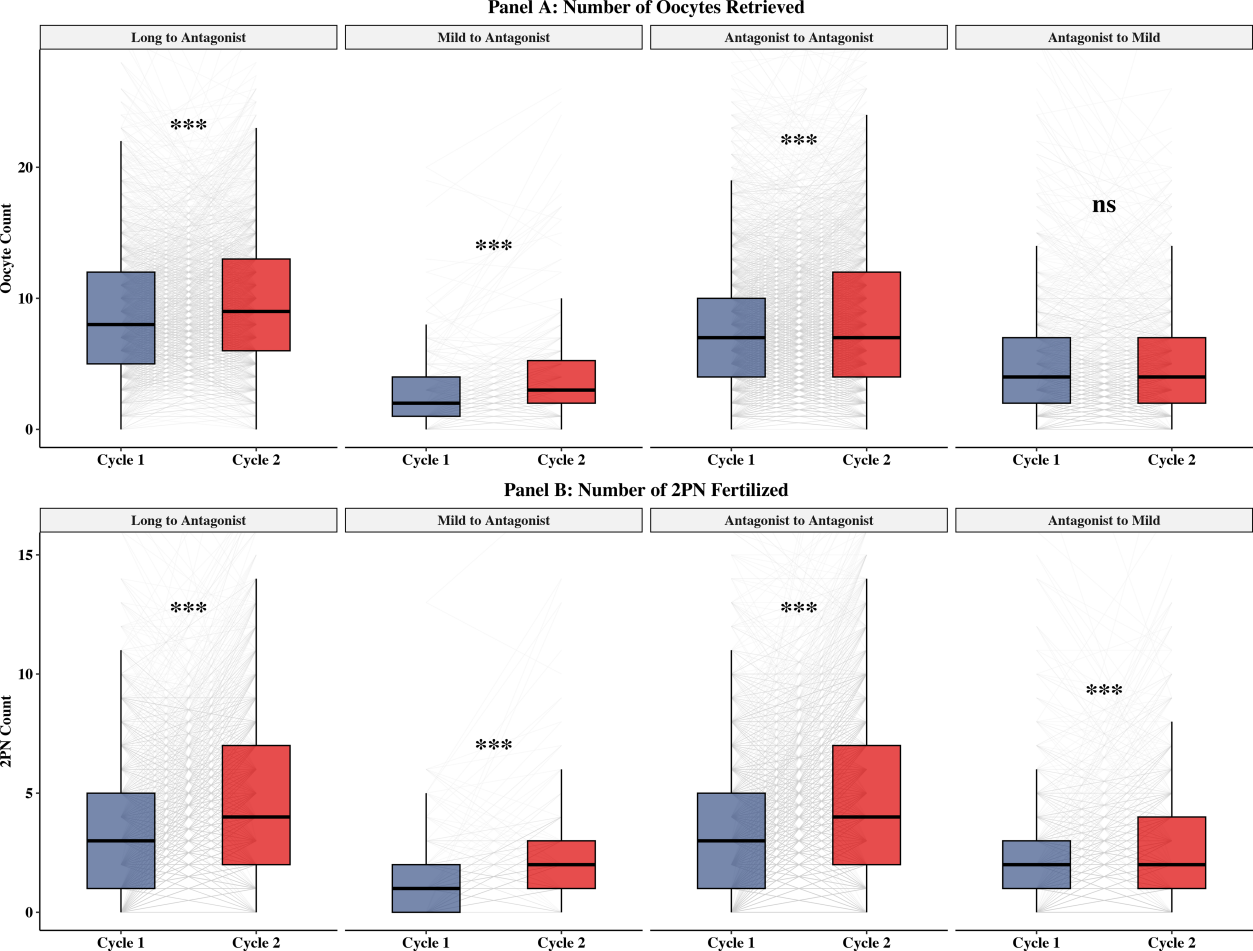
Comparison of laboratory outcomes between Cycle 1 and Cycle 2. **(A)** Median number of oocytes retrieved. **(B)** Median number of two-pronuclear (2PN) zygotes. The box-and-whisker plots represent the median (central line), interquartile range (box), and range (whiskers). ***P < 0.001; ns, non-significant (Wilcoxon signed-rank test).

**Figure 5:**
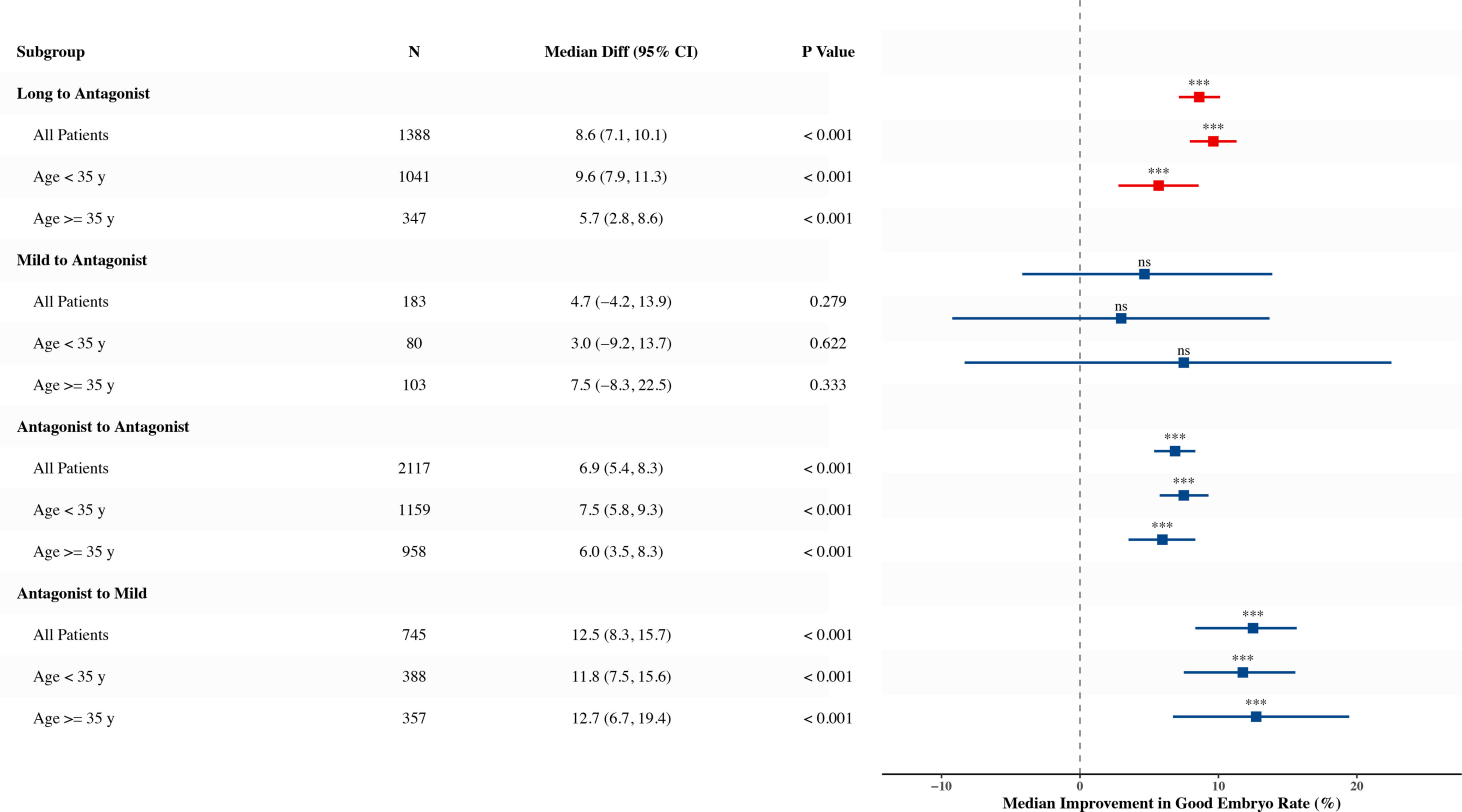
Subgroup analysis of the improvement in good-quality embryo rate. The forest plot displays the median difference (Cycle 2 minus Cycle 1) with 95% confidence intervals (CI) for each protocol transition group. Analyses were stratified by maternal age (<35 years and ≥35 years). Note that significant improvements were observed even in advanced age patients (≥35 y) for the Long and Antagonist transition groups. “***” indicates P < 0.001 (Wilcoxon signed-rank test).

Quality Rescue (Antagonist-to-Mild): This strategy did not result in increased oocyte numbers (Median: 4.0 vs 4.0, P = 0.642, **Figure 4A**). However, it achieved the most significant improvement in embryo competence, with the Good Embryo Rate (GER) rising from a median of 0.0% (0.0–25.0%) to 16.7% (0.0–39.6%) (P < 0.001, **Table 2**, **Figure 5**). The 2PN fertilization rate also improved significantly in this group (66.7% vs 75.0%, P < 0.001) (**Table 2**, **Supplementary Figure 2**).

Natural Variation (Reference): The Antagonist-to-Antagonist group also showed significant improvements in oocyte yield [7.0 (4.0–10.0) vs 7.0 (4.0–12.0), P < 0.001] and GER (12.5% vs 20.0%, P < 0.001) despite repeating the same protocol (**Table 2**, **Figure 4**).

### Clinical outcomes and safety

**Figure 6:**
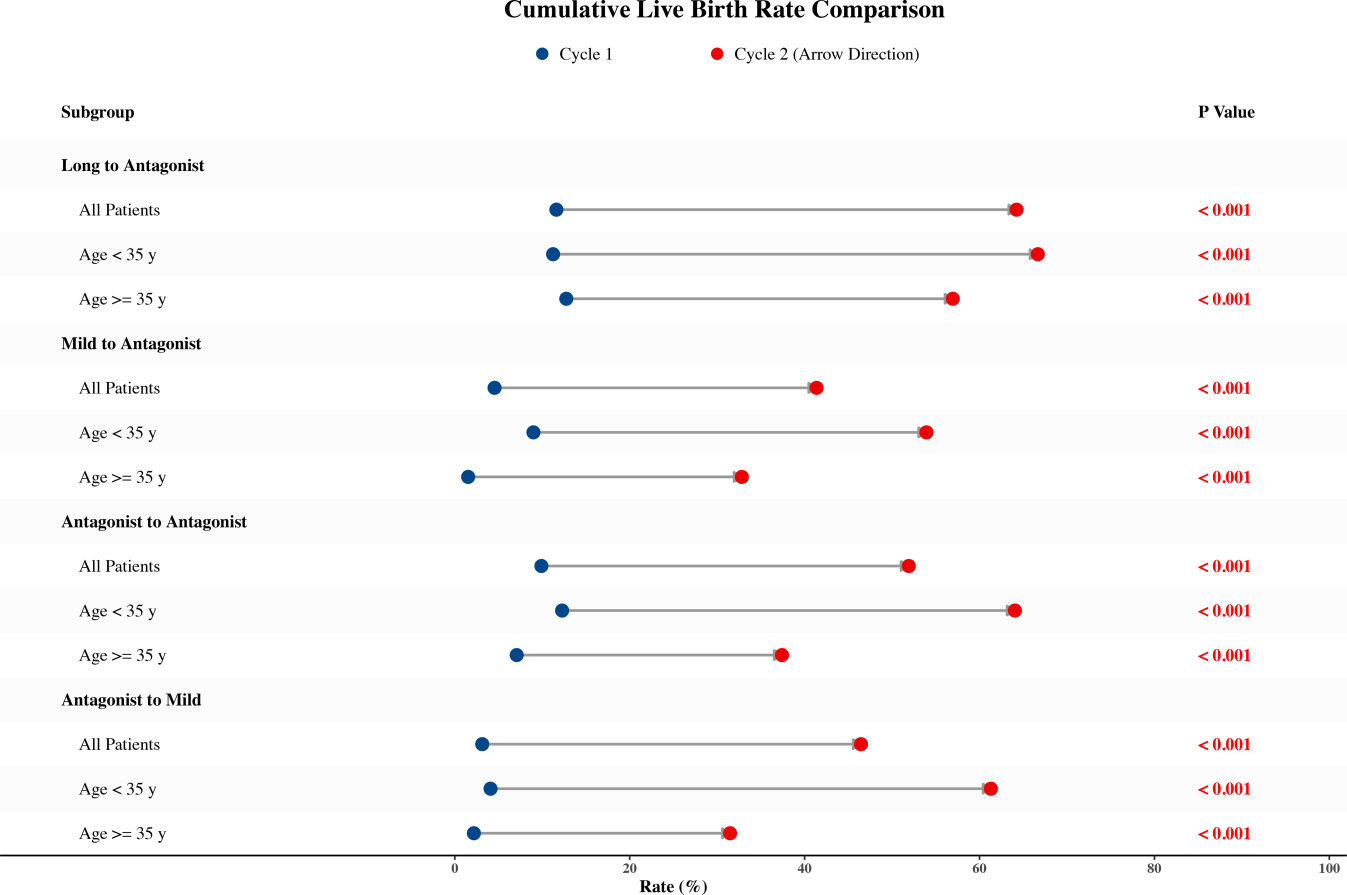
Dumbbell plot of live birth rate (LBR) improvement. The left end of each dumbbell represents the LBR in Cycle 1, and the right end (arrowhead) represents Cycle 2. The chart demonstrates consistent and significant improvements across all groups and age categories (<35 years and ≥35 years). *P*-values indicate the significance of the difference between cycles (McNemar’s test).

The primary outcome, Cumulative Live Birth Rate (CLBR), achieved significant increases across all four pathways in Cycle 2 (P < 0.001 for all) (**Figure 6**). The Long-to-Antagonist group reached the highest CLBR (64.2%), followed by the reference group (51.9%). Even in the poor-prognosis Mild-to-Antagonist group, CLBR increased nearly ten-fold from 4.5% to 41.4% (P < 0.001) (**Supplementary Figure 3**).

Regarding safety, miscarriage rates remained comparable between cycles for most groups (P > 0.05), and no cases of moderate or severe OHSS were recorded during the rescue cycles (**Table 2**).

### Predictors of cumulative live birth

**Figure 7:**
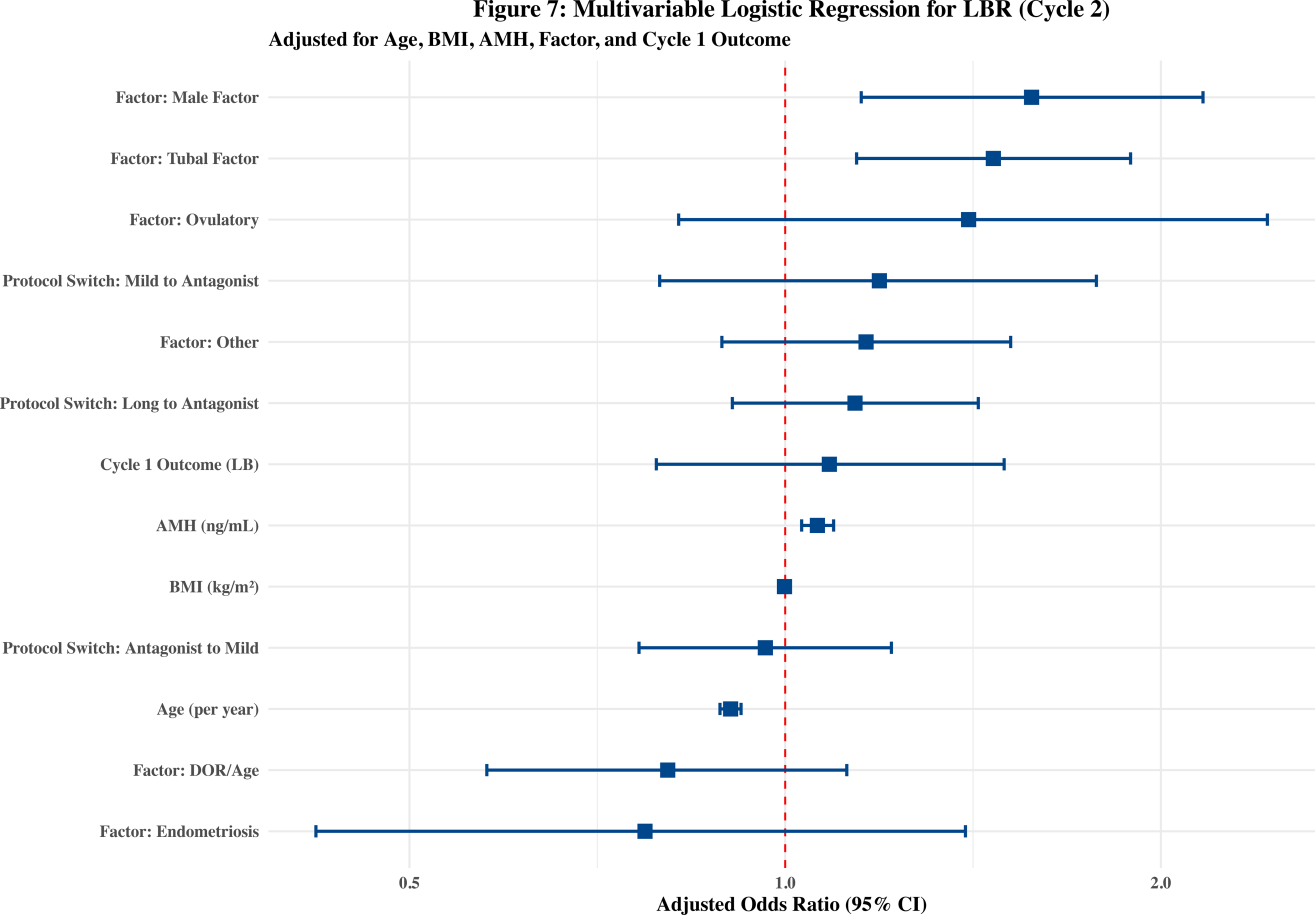
Multivariable logistic regression analysis for live birth rate in the second cycle. The “Antagonist to Antagonist” group served as the reference. Points represent the adjusted odds ratios (adjusted OR), and error bars indicate 95% confidence intervals (CI). The model was adjusted for maternal age, BMI, AMH, infertility factors, and Cycle 1 outcome.

Multivariable logistic regression demonstrated acceptable discriminative ability (AUC = 0.68, 95% CI: 0.66–0.71). Although the Hosmer-Lemeshow test yielded a P = 0.007, this is likely an artifact of high statistical power in a large sample size (n=4,632) rather than poor model calibration (**Table 3**, **Figure 7**). 1) Biological Constraints: Maternal age remained a significant negative predictor (Adjusted OR = 0.90, 95% CI: 0.89–0.92, P < 0.001), while AMH was positively associated with success (Adjusted OR = 1.06, 95% CI: 1.03–1.09, P < 0.001). 2) Etiological Factors: Male factor (Adjusted OR = 1.58, P = 0.005) and tubal factor (Adjusted OR = 1.47, P = 0.003) significantly increased the odds of achieving a live birth. 3) Protocol Impact: After adjusting for age and ovarian reserve, the specific protocol switch strategies (Long-to-Ant, Mild-to-Ant, Ant-to-Mild) did not demonstrate independent statistical significance (P > 0.05) relative to the reference group. This indicates that the observed clinical improvements were largely driven by the appropriate alignment of protocols with individual patient phenotypes rather than the inherent superiority of a specific transition.

**Table 3:** Multivariable logistic regression analysis of factors associated with live birth in the second cycle.

Variable	Adjusted OR (95% CI)	P value
Protocol Switch:		
Long to Antagonist	1.14 (0.91-1.43)	0.266
Mild to Antagonist	1.19 (0.79-1.78)	0.397
Antagonist to Mild	0.96 (0.76-1.22)	0.758
Age (per year)	0.90 (0.89-0.92)	< 0.001
BMI (kg/m²)	1.00 (0.99-1.01)	0.771
AMH (ng/mL)	1.06 (1.03-1.09)	< 0.001
Factor: DOR/Age	0.81 (0.58-1.12)	0.201
Factor: Endometriosis	0.77 (0.42-1.39)	0.396
Factor: Male Factor	1.58 (1.15-2.16)	0.005
Factor: Other	1.16 (0.89-1.52)	0.272
Factor: Ovulatory	1.40 (0.82-2.43)	0.220
Factor: Tubal Factor	1.47 (1.14-1.89)	0.003
Cycle 1 Outcome (LB)	1.08 (0.79-1.50)	0.618

The “Antagonist to Antagonist” protocol switch group served as the reference category. Adjusted odds ratios (adjusted OR) and 95% confidence intervals (CI) are presented. The model was adjusted for maternal age, BMI, baseline AMH, infertility factors, and cycle 1 outcome. *P* < 0.05 was considered statistically significant. OR, odds ratio; CI, confidence interval; BMI, body mass index; AMH, anti-Müllerian hormone; LB, live birth.

Model fit metrics: Area Under the Curve (AUC) = 0.68 (95% CI: 0.66-0.71); Hosmer-Lemeshow test P-value = 0.007.

## Discussion

In this large-scale paired-cycle analysis of 4,632 patients, we demonstrated that individualized management following an initial ART failure yields significant clinical improvements across diverse patient phenotypes.

First, our findings demonstrate that effective clinical rescue can be achieved through protocol optimization without the necessity of escalating Gn dosages. Traditionally, clinical practice has relied on escalating gonadotropin (Gn) dosages to rescue failed cycles, yet the benefits of such increments are frequently constrained by a “dose-response plateau”. In the present study, while maintaining strictly constant Gn dosages and stimulation durations across cycles, we observed a significant leap in CLBR across all four strategic pathways. This confirms that achieving an individualised environment match through either “strategic persistence” or “tactical switching” offers superior clinical efficacy and safety compared to simply increasing the medication burden.

Second, our data elucidate the multifaceted nature of the “rescue” mechanism. Within our phenotype-driven framework, we clearly distinguished between “quantity rescue”—aimed at expanding oocyte yield (e.g., transitioning to antagonist protocols)—and “quality rescue”—aimed at enhancing embryo competence (e.g., transitioning to mild stimulation).

Finally, by incorporating a reference group that repeated the index protocol, we quantified the substantial role of regression to the mean in ART outcomes. Specifically, the significant absolute increase in CLBR (from 9.9% to 51.9%) observed in the Antagonist-to-Antagonist group serves as the quantitative baseline proxy for natural biological variation, demonstrating that a substantial proportion of the clinical success achieved in the second attempt is driven by this inherent phenomenon rather than the protocol switch alone.

### Rationale for strategy selection

To systematically evaluate the efficacy of various protocol transition pathways, this study constructed a comprehensive decision matrix covering core “failure phenotypes.”

First, we established a robust biological benchmark. The Antagonist-to-Antagonist cohort was selected as the “Baseline Strategy.” By maintaining identical intervention logic across two consecutive cycles, we aimed to objectively reveal the cumulative benefits derived from “inter-cycle variability” and “regression to the mean.” This benchmark serves as a scientific yardstick to quantify the “additional therapeutic increment” provided by other transition pathways, preventing the over-attribution of success solely to protocol switching.

Second, the remaining three pathways were specifically tailored to the most common clinical failure scenarios: 1) Long-to-Antagonist (Unshackling Suppression): Targeted at patients exhibiting “over-suppression” or poor synchronization under GnRH agonists in Cycle 1, exploring the potential for improved recruitment after releasing the pituitary inhibition. 2) Mild-to-Antagonist (Quantity Rescue): Targeted at poor-prognosis patients with previous “recruitment failure” (≤3 oocytes), validating the feasibility of achieving a quantitative leap through escalated stimulation intensity. 3) Antagonist-to-Mild (Quality Rescue): Targeted at patients characterized by “compromised embryo quality” or abnormal fertilization, testing the optimization effect of a “less is more” approach in reducing ovarian metabolic load and enhancing oocyte cytoplasmic maturation.

Finally, the study design mirrors real-world clinical practice. Pathways such as Long-to-Long, which are rarely repeated in actual practice due to “risk-aversion preferences,” were intentionally excluded. This approach ensures that our cohorts reflect the natural decision-making tendencies of clinicians following an initial failure. By aligning the study design with clinical operational logic and strictly limiting the inter-cycle interval to within 12 months, we ensured high ecological validity and minimized the confounding impact of biological aging on our self-controlled analysis.

### Mechanisms of rescue: quantity vs. quality

Our results reveal that clinical success in the second cycle is not a monolithic phenomenon but is achieved through two distinct biological pathways depending on the failure phenotype (**Table 2**).

Quantity-Driven Pathway (Mild-to-Antagonist): For patients with previous “recruitment failure,” the significant rise in CLBR (4.5% to 41.4%) was primarily driven by a significant increase in the mean number of oocytes retrieved (Median 2.0 to 3.0, P < 0.001). Notably, their fertilization rate and Good Embryo Rate (GER) remained stable without significant divergence (P > 0.05). This confirms that for this phenotype, success depends predominantly on expanding the oocyte pool to compensate for low retrieval counts (16). While mild stimulation is advocated for its patient-friendly profile, our data suggest that for patients facing ‘time-to-pregnancy’ urgency, the Mild-to-Antagonist switch offers a pragmatic strategy to maximize the oocyte yield per retrieval cycle, thereby improving the cumulative probability of live birth within a shorter timeframe.

Quality-Rescue Pathway (Antagonist-to-Mild): For patients characterized by “embryo competence failure,” the Antagonist-to-Mild strategy significantly improved clinical outcomes without increasing the absolute number of oocytes retrieved (Median 4.0 vs 4.0, P = 0.642). This “quality rescue” was evidenced by the marked elevation in the Good Embryo Rate (Median 0.0% to 16.7%, P < 0.001). These findings support the “less is more” hypothesis, suggesting that for patients with compromised embryo quality, a milder approach may optimize oocyte nuclear-cytoplasmic maturation by avoiding the deleterious effects of high-dose FSH on the follicular microenvironment (17, 18) and preserving oocyte developmental potential which might otherwise be impaired by intensive stimulation (19, 20). This distinction is crucial; previous intraindividual comparisons between GnRH agonist and antagonist cycles failed to find significant differences in embryo morphokinetics, suggesting that merely switching the downregulation method is insufficient to alter embryo competency (21). In contrast, our findings highlight that reducing the gonadotropin load (via Mild stimulation)—rather than simply swapping analogs—is the key determinant for quality rescue.

### Impact of age stratification on protocol switching

Our subgroup analyses (**Figures 5**, **6**) revealed that patients of advanced maternal age (≥ 35 years) continued to derive significant clinical benefits from phenotype-driven protocol switching. For these older patients, diminished ovarian reserve and age-related decline in oocyte quality make them particularly vulnerable to the metabolic stress of intensive stimulation. Consequently, transitioning to a Mild protocol (Quality Rescue) effectively alleviates the deleterious effects of high-dose FSH on the sensitive aging follicular microenvironment, whereas switching to an Antagonist protocol (Quantity Rescue) maximizes the retrieval of the remaining, shrinking follicular cohort. This underscores that targeted intervention based on physiological constraints is arguably more critical for older patients than for their younger counterparts.

### “Unshackling” from over-suppression: the long-to-antagonist transition

The significant improvement in the Long-to-Antagonist group underscores the importance of oocyte cohort synchronization and the avoidance of pituitary over-suppression. 1)Physiological Environment: In Cycle 1, these patients underwent the GnRH agonist long protocol, which effectively depletes the pituitary of gonadotrophins but may lead to excessive suppression of endogenous LH and prolonged FSH stimulation. The transition to an antagonist protocol in Cycle 2 allows for a more physiological rise in early follicular FSH and avoids the “flare effect” and subsequent profound suppression. Furthermore, the transition to an antagonist protocol may provide a more favorable endocrine milieu by preserving endogenous LH pulsatility, which is often blunted in long agonist protocols. This ‘LH rescue’ effect could be particularly beneficial for patients with specific gonadotropin response genotypes (e.g., LHCGR polymorphisms) (22), although this requires further pharmacogenetic validation. 2)Impact on Recruitment: Our data show that this switch significantly increased oocyte retrieval and fertilization rates. This suggests that for certain patients, the profound suppression of the long protocol may have impaired follicular recruitment or early development (23).

Clinical Significance: The substantial rise in CLBR from 11.6% to 64.2% in this group —the highest among all switch pathways—indicates that “unshackling” the ovaries from intensive down-regulation can restore the developmental potential of the oocyte cohort in patients previously sensitive to over-suppression (24).

### The statistical paradox: precision matching vs. independent superiority

A pivotal finding of our study is the apparent “statistical paradox” in the multivariable logistic regression (**Table 3**): while clinical outcomes significantly improved across all pathways, the specific protocol switch strategies did not emerge as independent predictors of live birth. The absolute increase in CLBR in the Reference group (9.9% to 51.9%) serves as a conceptual baseline proxy for regression to the mean. It is critical to note that since clinical triage was phenotype-driven, baseline differences between groups were expected. The regression model (where switching was not an independent predictor) confirms that protocol switching is not a ‘magic bullet’ overriding patient biology. Instead, it acts as a mediator aligning interventions with the patient’s specific physiological constraints (Age, AMH). We believe this reflects the “triage effect” inherent in precision medicine. In clinical practice, physicians “triage” patients based on failure phenotypes—directing those with recruitment failure toward “quantity rescue” and those with poor embryo quality toward “quality rescue.”

Our findings refine the conclusions of previous studies. For instance, previous research reported that in normal responders, switching between agonist and antagonist protocols following a failed ICSI cycle offered no clinical benefit over repeating the same protocol (25). While their conclusion supports our observation of the “repetition effect” in the reference group, their lack of stratification masked the benefits of protocol switching for specific phenotypes. By treating the population as a monolith, previous studies may have “diluted” the therapeutic effects of switching. In contrast, our study demonstrates that when patients are “triaged” based on specific failure causes—such as moving recruitment-failure patients to high-dose antagonists (Mild-to-Ant)—protocol switching yields a statistically significant advantage. This confirms that the core question is not whether to switch, but how to match the switch to the patient’s biological constraints.

This aligns with recent evidence utilizing propensity score matching (PSM), which demonstrates that protocol superiority is strictly phenotype-dependent (26). Specifically, external data suggest that while the GnRH agonist long protocol significantly favors older women (≥30 years) with moderate ovarian reserve, it may actually be detrimental (OR 0.54) for older patients with both obesity (BMI ≥ 24 kg/m^2) and low AMH (≤3ng/ml). This underscores that the lack of independent significance for protocol switching in our model is because success is driven by the accuracy of the match between the strategy and the patient’s specific age, BMI, and AMH profile.

Ultimately, our regression analysis confirms that maternal age (aOR 0.90) and AMH (aOR 1.06) remain the absolute determinants of ART success. This finding highlights a fundamental biological reality: although our self-controlled design eliminates inter-individual heterogeneities, it cannot circumvent the intrinsic decay of oocyte quality. Age-related aneuploidy and mitochondrial dysfunction remain irreversible biological barriers that no stimulation protocol can fully remediate (27, 28). Furthermore, the predictive power of AMH explains why the Mild-to-Antagonist pathway achieved a “probabilistic reversal”; patients with higher AMH typically possess a wider “recruitment window,” allowing the second cycle to expand the oocyte pool (from 3.1 to 4.6) and thus increasing the statistical probability of obtaining a euploid embryo (29–31). Thus, the success of a second cycle is a synergy between “accurate tactical adjustment” and the “inherent biological reserve” of the patient. This serves as a crucial reminder for clinicians: before attributing success to a ‘magic’ protocol change, we must first acknowledge the baseline probability of success driven by natural physiological variation. Over-intervention in the second cycle may not only be unnecessary but could also incur avoidable financial and physical burdens.

The primary strength of this study lies in its massive scale and the rigorous self-controlled design. By including 4,632 paired cycles, we effectively neutralized the confounding impact of inter-individual heterogeneity, such as genetic background and baseline lifestyle factors, which often plague traditional cohort studies. Furthermore, the inclusion of a “repetition reference group” provides one of the most robust quantifications of the “regression to the mean” phenomenon in current ART literature, offering a necessary corrective to the over-interpretation of protocol switching.

Despite these strengths, several limitations warrant consideration. First, the retrospective nature introduces inherent selection bias and confounding by indication, as clinicians selected protocols based on prior failure. While our self-controlled design mitigates inter-individual variability, it cannot fully eliminate intra-individual confounding over time. Second, while we controlled for biological age and AMH, we could not account for potential “inter-cycle drift”—such as unrecorded lifestyle modifications, psychological stress fluctuations, or the use of adjuvant therapies (e.g., DHEA, CoQ10) between attempts. Finally, this was a single-Centre study conducted at a high-volume fertility clinic. While this ensured consistency in laboratory standards and clinical SOPs, the findings should be extrapolated to Centres with differing laboratory environments or patient demographics with caution.

## Conclusions

In conclusion, our study provides a robust, evidence-based framework for the management of ART failure. We demonstrate that while individualised protocol switching is a powerful tool for “quantity” or “quality” rescue, its clinical utility must be understood within the context of inherent physiological variability. Clinicians should reassure patients that a failed first cycle is not a definitive verdict on their reproductive potential. Often, the path to success lies in physiological normalization and the strategic alignment of clinical protocols with individual biological phenotypes.

## Data Availability

The raw data supporting the conclusions of this article will be made available by the authors, without undue reservation.

## Supplementary material

Supplementary Figure 1 Distribution of primary reasons for failure in the first *in vitro* fertilization (IVF) cycle. Patients were categorized by their protocol transition group. The stacked bars represent the proportion of failures attributed to low oocyte yield (≤3), fertilization failure, lack of good-quality embryos, or implantation/developmental failure.

Supplementary Figure 2 Paired comparison of fertilization and good-quality embryo rates. The left panel displays the 2PN fertilization rate (2PN count/oocytes retrieved), and the right panel shows the good-quality embryo rate (good-quality embryos/oocytes retrieved). P-values were calculated using the Wilcoxon signed-rank test.****P* < 0.001; **P* < 0.05; ns, non-significant.

Supplementary Figure 3 Dumbbell plot of Clinical Pregnancy Rate (CPR) improvement. Comparison of CPR between Cycle 1 and Cycle 2, stratified by maternal age. *P*-values were calculated using McNemar’s test.
