# Supplementary material for: Phenotype-driven protocol switching is associated with improved ART outcomes under constant gonadotropin dosage: a self-controlled analysis of 4,632 cycles
Source: Front Endocrinol (Lausanne). 2026 May 13;17:1816340. doi: 10.3389/fendo.2026.1816340 (PMC13212103; doi:10.3389/fendo.2026.1816340)
Supplement: Supplementary Figure 1 — Distribution of primary reasons for failure in the first in vitro fertilization (IVF) cycle. Patients were categorized by their protocol transition group. The stacked bars represent the proportion of failures attributed to low oocyte yield (≤3), fertilization failure, lack of good-quality embryos, or implantation/developmental failure. [file DataSheet1.pdf]

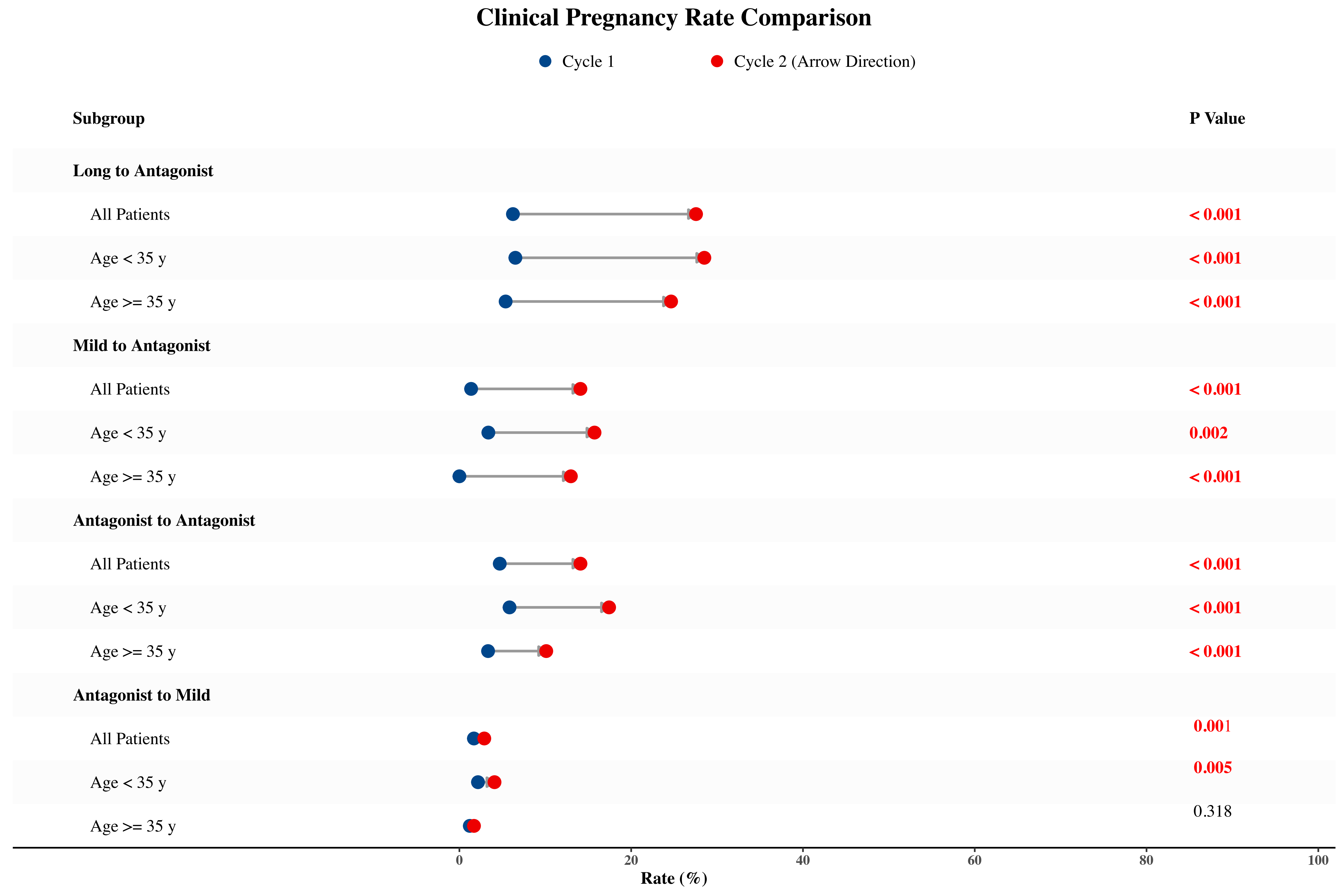
Clinical Pregnancy Rate Comparison
Cycle 1
Cycle 2 (Arrow Direction)
Subgroup
P Value
Long to Antagonist
All Patients
< 0.001
Age < 35 y
< 0.001
Age >= 35 y
< 0.001
Mild to Antagonist
All Patients
< 0.001
Age < 35 y
0.002
Age >= 35 y
< 0.001
Antagonist to Antagonist
All Patients
< 0.001
Age < 35 y
< 0.001
Age >= 35 y
< 0.001
Antagonist to Mild
All Patients
0.001
Age < 35 y
0.005
Age >= 35 y
0.318
0
20
40
60
80
100
Rate (%)
