# Supplementary figures and images for: Phenotype-driven protocol switching is associated with improved ART outcomes under constant gonadotropin dosage: a self-controlled analysis of 4,632 cycles
Source: Front Endocrinol (Lausanne). 2026 May 13;17:1816340. doi: 10.3389/fendo.2026.1816340 (PMC13212103; doi:10.3389/fendo.2026.1816340)

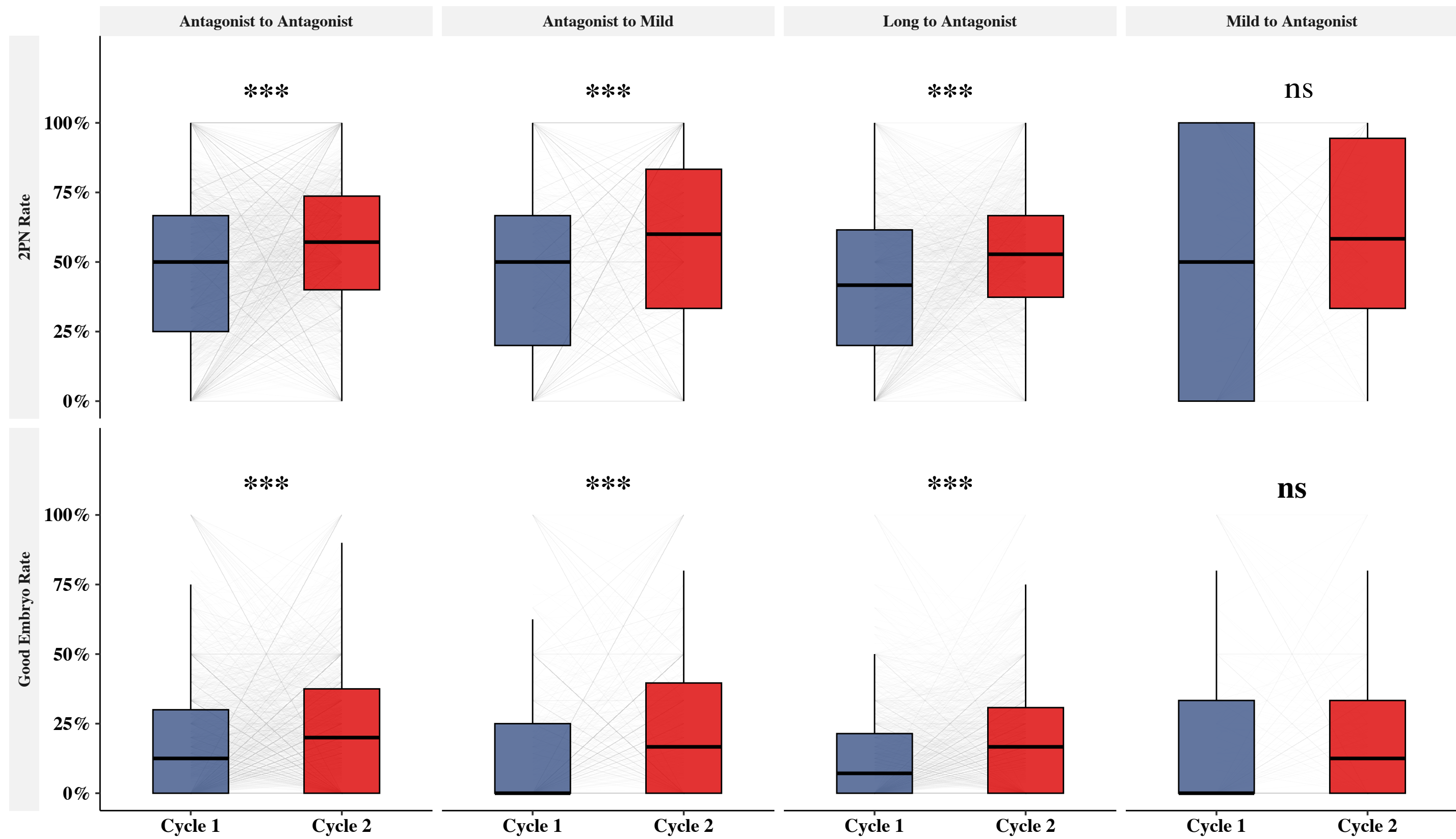

Supplement: Supplementary Figure 2 — Paired comparison of fertilization and good-quality embryo rates. The left panel displays the 2PN fertilization rate (2PN count/oocytes retrieved), and the right panel shows the good-quality embryo rate (good-quality embryos/oocytes retrieved). P-values were calculated using the Wilcoxon signed-rank test.***P < 0.001; *P < 0.05; ns, non-significant. [file DataSheet2.pdf]

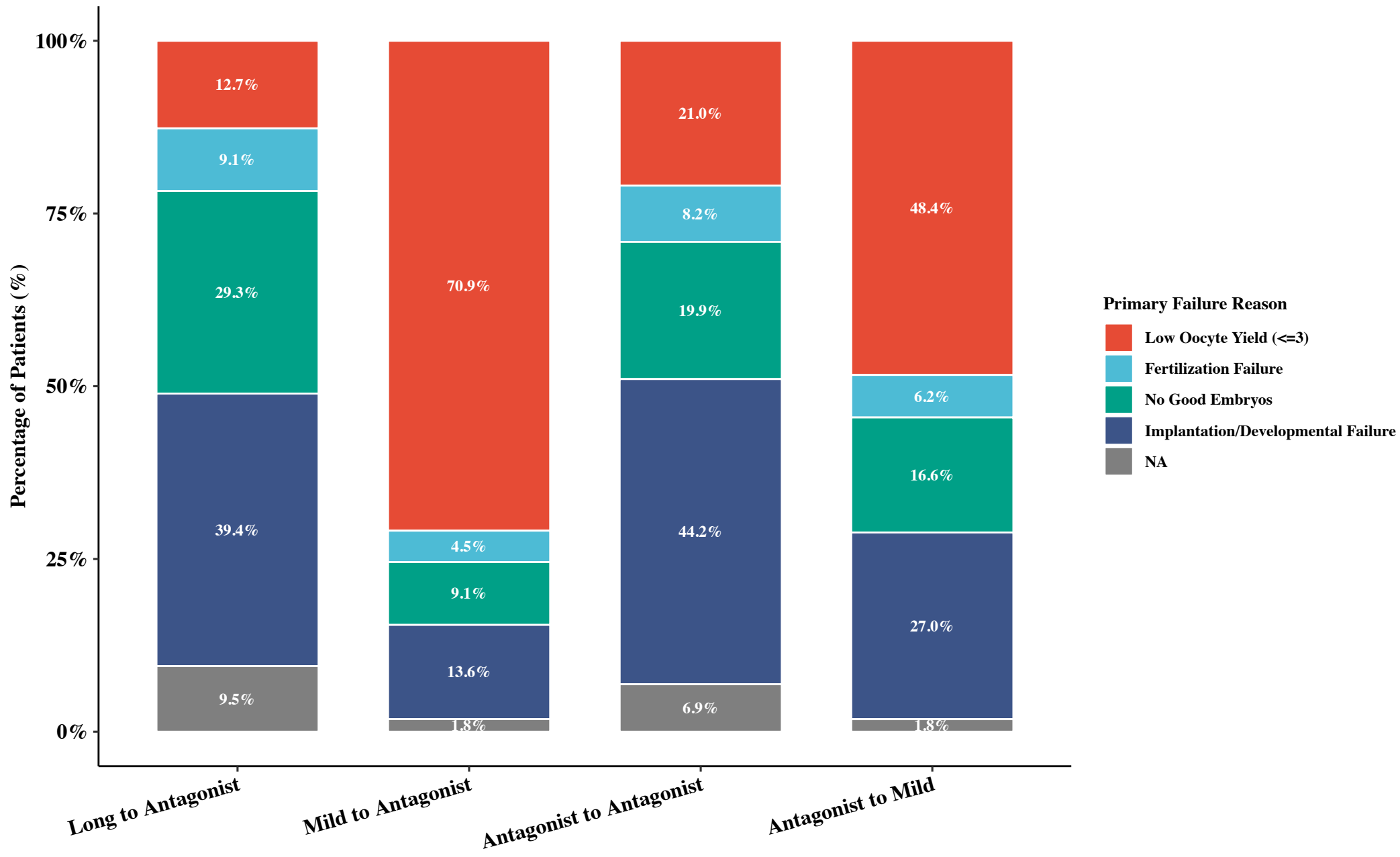

Supplement: Supplementary Figure 3 — Dumbbell plot of Clinical Pregnancy Rate (CPR) improvement. Comparison of CPR between Cycle 1 and Cycle 2, stratified by maternal age. P-values were calculated using McNemar’s test. [file DataSheet3.pdf]
